# Supplementary material for: A Combined Proteomics, Metabolomics and In Vivo Analysis Approach for the Characterization of Probiotics in Large-Scale Production
Source: Biomolecules. 2020 Jan 18;10(1):157. doi: 10.3390/biom10010157 (PMC7022454; doi:10.3390/biom10010157)
Supplement: Supplementary file 1 [file biomolecules-10-00157-s001.zip › biomolecules-666446--SUPPL/Figure S1.docx]

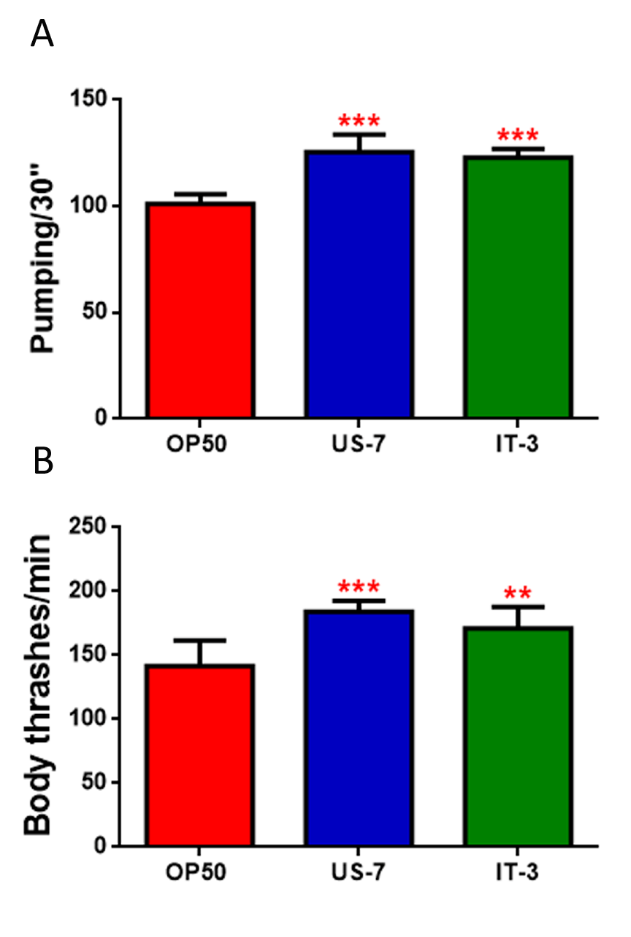


**Figure 1.** Analysis of aging markers analysis of wild type nematodes fed US-7 or IT-3. (A) Pumping rate of 13-day-old worms, measured for 30 seconds and determined from the mean of 10 worms for each bacterial strain. Worms fed OP50 were used as control (***p<0.001). (B) Body bend frequency of *C. elegans* fed with US-7 or IT-3 with respect to OP50. Worms were measured for 30 seconds. Statistical analysis was evaluated by one-way ANOVA with the Bonferroni posttest; asterisks indicate significant differences (**p<0.01; ***p<0.001).
