# Supplementary material for: A Combined Proteomics, Metabolomics and In Vivo Analysis Approach for the Characterization of Probiotics in Large-Scale Production
Source: Biomolecules. 2020 Jan 18;10(1):157. doi: 10.3390/biom10010157 (PMC7022454; doi:10.3390/biom10010157)
Supplement: Supplementary file 1 [file biomolecules-10-00157-s001.zip › biomolecules-666446--SUPPL/Table S1.docx]

**Tables**

**Table S1.** Probiotic products: details and manufacturing origin

| **Code** | **lot number** | **expiration date (month/year)** | **Production site** |
| --- | --- | --- | --- |
| IT-3 ^*^ ^#^ | 608008 | 08/2018 | Nutrilinea/CSL, Italy |
| IT-2 | 607006 | 07/2018 | Nutrilinea/CSL, Italy |
| IT-1 | 605001 | 05/2018 | Nutrilinea/CSL, Italy |
| US-4 | 10162797 | 10/2018 | Danisco/DuPont, USA |
| US-7 ^*#^ | 1634022 | 06/2018 | Danisco/DuPont, USA |
| US-2 | 1615803 | 12/2017 | Danisco/DuPont, USA |
| US-6 ^*^ | DM019 | 06/2017 | Danisco/DuPont, USA |
| US-1e | JL789 | 01/2016 | Danisco/DuPont, USA |
| US-5e | 0238622 | 04/2015 | Danisco/DuPont, USA |
| US-3e | LJ120 | 01/2014 | Danisco/DuPont, USA |

^*^Samples not analysed by metabolomics

^#^Samples tested in *Caenorhabditis elegans*
