# Supplementary material for: A Combined Proteomics, Metabolomics and In Vivo Analysis Approach for the Characterization of Probiotics in Large-Scale Production
Source: Biomolecules. 2020 Jan 18;10(1):157. doi: 10.3390/biom10010157 (PMC7022454; doi:10.3390/biom10010157)
Supplement: Supplementary file 1 [file biomolecules-10-00157-s001.zip › biomolecules-666446--SUPPL/Table S10.docx]

**Table S10. Median lifespan and 50% viability in worms fed OP50, US-7 or IT-3 products.**

|  | Median lifespan | 50% viability |
| --- | --- | --- |
| OP50 | 18 ± 1.1 | 11 ± 0.7 |
| US-7 | 24 ± 1.2 | 17 ± 0.5 |
| IT-3 | 19 ± 0.5 | 16 ± 0.6 |
