# Supplementary material for: A Combined Proteomics, Metabolomics and In Vivo Analysis Approach for the Characterization of Probiotics in Large-Scale Production
Source: Biomolecules. 2020 Jan 18;10(1):157. doi: 10.3390/biom10010157 (PMC7022454; doi:10.3390/biom10010157)
Supplement: Supplementary file 1 [file biomolecules-10-00157-s001.zip › biomolecules-666446--SUPPL/Table S3.docx]

**Table S3.** Summary of the differentially abundant proteins detected between each Italy-made product and the US-4, and between each US-product and the US-4.

|  | # proteins with increased abundance | # proteins with decreased abundance | Expiry date | Manufacturing site |
| --- | --- | --- | --- | --- |
| IT-2 vs US-4 | 583 | 804 | 07/2018 vs 10/2018 | Italy-made vs US-made |
| IT-3 vs US-4 | 547 | 574 | 08/2018 vs 10/2018 | Italy-made vs US-made |
| IT-1 vs US-4 | 579 | 451 | 05/2018 vs 10/2018 | Italy-made vs US-made |
| US3e vs US-4 | 378 | 291 | 01/2014 vs 10/2018 | US-made |
| US-5e vs US-4 | 132 | 375 | 04/2015 vs 10/2018 | US-made |
| US-1e vs US-4 | 55 | 68 | 01/2016 vs 10/2018 | US-made |
| US-6 vs US-4 | 49 | 26 | 06/2017 vs 10/2018 | US-made |
| US-2 vs US-4 | 10 | 9 | 12/2017 vs 10/2018 | US-made |
| US-7 vs US-4 | 127 | 50 | 06/2018 vs 10/2018 | US-made |
