# Supplementary material for: A Combined Proteomics, Metabolomics and In Vivo Analysis Approach for the Characterization of Probiotics in Large-Scale Production
Source: Biomolecules. 2020 Jan 18;10(1):157. doi: 10.3390/biom10010157 (PMC7022454; doi:10.3390/biom10010157)
Supplement: Supplementary file 1 [file biomolecules-10-00157-s001.zip › biomolecules-666446--SUPPL/Table S4_STRING Enrichment analysis L.casei.docx]

**Table S4:** STRING net statistics output and GO and KEGG pathway annotation enrichment analysis obtained by processing proteins detected more abundant in *Lactobacillus paracasei* from IT-preparations. *Lactobacillus casei* was set as reference organism. PPI and GO/KEGG annotation enrichments were retained significant with a FDR p < 0.001 and p < 0.005 (not shadowed area), respectively.

**Net statistics output**

| number of nodes: | 565 |
| --- | --- |
| number of edges: | 3120 |
| average node degree: | 11 |
| avg. local clustering coefficient: | 0.41 |
| expected number of edges: | 2120 |
| PPI enrichment p-value: | < 1.0e-16 |

**GO BP**

| **Pathway ID** | **Pathway description** | **Count in gene set** | **False discovery rate** |
| --- | --- | --- | --- |
| GO:0034641 | cellular nitrogen compound metabolic process | 111 | 4.17e-39 |
| GO:0006807 | nitrogen compound metabolic process | 114 | 1.48e-38 |
| GO:0008150 | biological_process | 133 | 1.48e-38 |
| GO:0008152 | metabolic process | 129 | 2.87e-38 |
| GO:0044271 | cellular nitrogen compound biosynthetic process | 89 | 4.33e-38 |
| GO:0043170 | macromolecule metabolic process | 97 | 5.62e-38 |
| GO:0044260 | cellular macromolecule metabolic process | 95 | 5.62e-38 |
| GO:0009987 | cellular process | 126 | 6.78e-38 |
| GO:0044237 | cellular metabolic process | 123 | 6.78e-38 |
| GO:0044238 | primary metabolic process | 123 | 6.78e-38 |
| GO:0071704 | organic substance metabolic process | 126 | 6.78e-38 |
| GO:0034645 | cellular macromolecule biosynthetic process | 76 | 1.25e-35 |
| GO:0010467 | gene expression | 75 | 1.83e-34 |
| GO:0044249 | cellular biosynthetic process | 98 | 2.4e-34 |
| GO:1901576 | organic substance biosynthetic process | 99 | 2.73e-34 |
| GO:0019538 | protein metabolic process | 69 | 5.83e-34 |
| GO:1901564 | organonitrogen compound metabolic process | 88 | 1.54e-32 |
| GO:0044267 | cellular protein metabolic process | 65 | 1.59e-32 |
| GO:0006412 | translation | 59 | 8.88e-32 |
| GO:1901566 | organonitrogen compound biosynthetic process | 83 | 3.5e-31 |
| GO:0006139 | nucleobase-containing compound metabolic process | 62 | 1.4e-21 |
| GO:0006725 | cellular aromatic compound metabolic process | 63 | 8.53e-17 |
| GO:0046483 | heterocycle metabolic process | 63 | 8.53e-17 |
| GO:1901360 | organic cyclic compound metabolic process | 63 | 1.47e-16 |
| GO:0090304 | nucleic acid metabolic process | 38 | 2.04e-12 |
| GO:0044763 | single-organism cellular process | 59 | 2.6e-12 |
| GO:0044699 | single-organism process | 60 | 2.84e-11 |
| GO:0034654 | nucleobase-containing compound biosynthetic process | 29 | 1.31e-10 |
| GO:0044710 | single-organism metabolic process | 53 | 7.96e-10 |
| GO:1901135 | carbohydrate derivative metabolic process | 26 | 1.1e-09 |
| GO:0016070 | RNA metabolic process | 27 | 1.6e-09 |
| GO:0055086 | nucleobase-containing small molecule metabolic process | 24 | 5.94e-09 |
| GO:0072521 | purine-containing compound metabolic process | 21 | 7.15e-09 |
| GO:0044281 | small molecule metabolic process | 40 | 1.11e-08 |
| GO:1901137 | carbohydrate derivative biosynthetic process | 22 | 1.27e-08 |
| GO:0009117 | nucleotide metabolic process | 22 | 7.02e-08 |
| GO:0006163 | purine nucleotide metabolic process | 19 | 9.48e-08 |
| GO:0009259 | ribonucleotide metabolic process | 19 | 9.48e-08 |
| GO:0009150 | purine ribonucleotide metabolic process | 18 | 1.33e-07 |
| GO:0009260 | ribonucleotide biosynthetic process | 16 | 2.33e-07 |
| GO:0072522 | purine-containing compound biosynthetic process | 16 | 2.33e-07 |
| GO:0009152 | purine ribonucleotide biosynthetic process | 15 | 2.87e-07 |
| GO:0009123 | nucleoside monophosphate metabolic process | 18 | 3.12e-07 |
| GO:0009165 | nucleotide biosynthetic process | 18 | 3.12e-07 |
| GO:0034660 | ncRNA metabolic process | 18 | 3.12e-07 |
| GO:0018130 | heterocycle biosynthetic process | 31 | 4e-07 |
| GO:0019438 | aromatic compound biosynthetic process | 31 | 4e-07 |
| GO:0050794 | regulation of cellular process | 19 | 4.64e-07 |
| GO:0019637 | organophosphate metabolic process | 23 | 5.79e-07 |
| GO:1901362 | organic cyclic compound biosynthetic process | 31 | 5.98e-07 |
| GO:0009124 | nucleoside monophosphate biosynthetic process | 15 | 7.7e-07 |
| GO:0009161 | ribonucleoside monophosphate metabolic process | 17 | 9.86e-07 |
| GO:0009167 | purine ribonucleoside monophosphate metabolic process | 16 | 1.43e-06 |
| GO:0006399 | tRNA metabolic process | 15 | 1.98e-06 |
| GO:0031323 | regulation of cellular metabolic process | 15 | 1.98e-06 |
| GO:0051171 | regulation of nitrogen compound metabolic process | 15 | 1.98e-06 |
| GO:0060255 | regulation of macromolecule metabolic process | 15 | 1.98e-06 |
| GO:0009156 | ribonucleoside monophosphate biosynthetic process | 14 | 2.76e-06 |
| GO:0009116 | nucleoside metabolic process | 16 | 3.23e-06 |
| GO:0009168 | purine ribonucleoside monophosphate biosynthetic process | 13 | 3.52e-06 |
| GO:0006418 | tRNA aminoacylation for protein translation | 10 | 4.39e-06 |
| GO:0042278 | purine nucleoside metabolic process | 15 | 4.47e-06 |
| GO:0080090 | regulation of primary metabolic process | 15 | 4.47e-06 |
| GO:0090407 | organophosphate biosynthetic process | 19 | 6.22e-06 |
| GO:0006796 | phosphate-containing compound metabolic process | 24 | 6.96e-06 |
| GO:0009119 | ribonucleoside metabolic process | 15 | 1.01e-05 |
| GO:0046128 | purine ribonucleoside metabolic process | 14 | 1.57e-05 |
| GO:2000112 | regulation of cellular macromolecule biosynthetic process | 13 | 2.31e-05 |
| GO:0042455 | ribonucleoside biosynthetic process | 12 | 3.35e-05 |
| GO:0046129 | purine ribonucleoside biosynthetic process | 11 | 4.75e-05 |
| GO:0010468 | regulation of gene expression | 12 | 8.1e-05 |
| GO:0044711 | single-organism biosynthetic process | 28 | 0.000179 |
| GO:0071840 | cellular component organization or biogenesis | 14 | 0.000251 |
| GO:0006417 | regulation of translation | 6 | 0.000306 |
| GO:0042777 | plasma membrane ATP synthesis coupled proton transport | 6 | 0.000306 |
| GO:0009141 | nucleoside triphosphate metabolic process | 10 | 0.000429 |
| GO:0006082 | organic acid metabolic process | 21 | 0.000533 |
| GO:0006259 | DNA metabolic process | 12 | 0.000638 |
| GO:0046034 | ATP metabolic process | 9 | 0.000638 |
| GO:0019752 | carboxylic acid metabolic process | 20 | 0.000837 |
| GO:0032774 | RNA biosynthetic process | 10 | 0.000879 |
| GO:0044085 | cellular component biogenesis | 11 | 0.00104 |
| GO:0009142 | nucleoside triphosphate biosynthetic process | 7 | 0.00129 |
| GO:0006189 | de novo IMP biosynthetic process | 5 | 0.00144 |
| GO:0006950 | response to stress | 10 | 0.00157 |
| GO:0006351 | transcription, DNA-templated | 9 | 0.0026 |
| GO:0019219 | regulation of nucleobase-containing compound metabolic process | 9 | 0.0026 |
| GO:0007049 | cell cycle | 6 | 0.00471 |
| GO:0051301 | cell division | 6 | 0.00471 |
| GO:0006281 | DNA repair | 9 | 0.00478 |
| GO:0006260 | DNA replication | 5 | 0.00621 |
| GO:0006414 | translational elongation | 4 | 0.00621 |
| GO:0010605 | negative regulation of macromolecule metabolic process | 4 | 0.00621 |
| GO:0043933 | macromolecular complex subunit organization | 4 | 0.00621 |
| GO:0045934 | negative regulation of nucleobase-containing compound metabolic process | 4 | 0.00621 |
| GO:0065008 | regulation of biological quality | 7 | 0.00648 |
| GO:0006520 | cellular amino acid metabolic process | 15 | 0.0108 |
| GO:0016043 | cellular component organization | 8 | 0.0128 |
| GO:0034470 | ncRNA processing | 8 | 0.0128 |
| GO:0006508 | proteolysis | 5 | 0.0162 |
| GO:0006810 | transport | 8 | 0.0205 |
| GO:0042254 | ribosome biogenesis | 6 | 0.0205 |
| GO:0055085 | transmembrane transport | 7 | 0.0212 |
| GO:0006298 | mismatch repair | 3 | 0.0265 |
| GO:0006415 | translational termination | 3 | 0.0265 |
| GO:0006450 | regulation of translational fidelity | 3 | 0.0265 |
| GO:0045892 | negative regulation of transcription, DNA-templated | 3 | 0.0265 |
| GO:0051052 | regulation of DNA metabolic process | 3 | 0.0265 |
| GO:0050793 | regulation of developmental process | 5 | 0.0311 |

**GO MF**

| **Pathway ID** | **Pathway description** | **Count in gene set** | **False discovery rate** |
| --- | --- | --- | --- |
| GO:0003674 | molecular_function | 131 | 1.83e-38 |
| GO:0005488 | binding | 103 | 6.49e-35 |
| GO:0097159 | organic cyclic compound binding | 93 | 9.82e-35 |
| GO:1901363 | heterocyclic compound binding | 93 | 9.82e-35 |
| GO:0003676 | nucleic acid binding | 57 | 2.63e-23 |
| GO:0003723 | RNA binding | 39 | 1.15e-18 |
| GO:0032549 | ribonucleoside binding | 51 | 1.15e-18 |
| GO:0043167 | ion binding | 61 | 1.55e-18 |
| GO:0000166 | nucleotide binding | 52 | 2.06e-18 |
| GO:0003735 | structural constituent of ribosome | 37 | 2.06e-18 |
| GO:0032550 | purine ribonucleoside binding | 50 | 2.06e-18 |
| GO:0032555 | purine ribonucleotide binding | 50 | 2.06e-18 |
| GO:0035639 | purine ribonucleoside triphosphate binding | 50 | 2.06e-18 |
| GO:0036094 | small molecule binding | 53 | 2.24e-18 |
| GO:0043168 | anion binding | 52 | 3.47e-18 |
| GO:0003824 | catalytic activity | 77 | 1.21e-17 |
| GO:0019843 | rRNA binding | 26 | 1.73e-14 |
| GO:0005524 | ATP binding | 40 | 2.3e-13 |
| GO:0016874 | ligase activity | 22 | 1.3e-10 |
| GO:0016787 | hydrolase activity | 32 | 4.45e-10 |
| GO:0017111 | nucleoside-triphosphatase activity | 15 | 2.73e-07 |
| GO:0016462 | pyrophosphatase activity | 16 | 6.18e-07 |
| GO:0005525 | GTP binding | 11 | 1.12e-06 |
| GO:0003677 | DNA binding | 17 | 5.11e-06 |
| GO:0004812 | aminoacyl-tRNA ligase activity | 10 | 5.11e-06 |
| GO:0016876 | ligase activity, forming aminoacyl-tRNA and related compounds | 11 | 5.28e-06 |
| GO:0000049 | tRNA binding | 10 | 2.37e-05 |
| GO:0046872 | metal ion binding | 22 | 3.65e-05 |
| GO:0008135 | translation factor activity, RNA binding | 7 | 7.59e-05 |
| GO:0046933 | proton-transporting ATP synthase activity, rotational mechanism | 6 | 0.000339 |
| GO:0003924 | GTPase activity | 7 | 0.000407 |
| GO:0016887 | ATPase activity | 7 | 0.000407 |
| GO:0016879 | ligase activity, forming carbon-nitrogen bonds | 9 | 0.000708 |
| GO:0000287 | magnesium ion binding | 11 | 0.00372 |
| GO:0016740 | transferase activity | 19 | 0.00726 |
| GO:0003746 | translation elongation factor activity | 4 | 0.0085 |
| GO:0004519 | endonuclease activity | 6 | 0.0136 |
| GO:0016884 | carbon-nitrogen ligase activity, with glutamine as amido-N-donor | 5 | 0.021 |
| GO:0070011 | peptidase activity, acting on L-amino acid peptides | 5 | 0.021 |
| GO:0016788 | hydrolase activity, acting on ester bonds | 9 | 0.0249 |
| GO:0004175 | endopeptidase activity | 4 | 0.0306 |
| GO:0002161 | aminoacyl-tRNA editing activity | 3 | 0.0366 |
| GO:0030983 | mismatched DNA binding | 3 | 0.0366 |
| GO:0046961 | proton-transporting ATPase activity, rotational mechanism | 3 | 0.0366 |
| GO:0008270 | zinc ion binding | 6 | 0.0445 |

**GO CC**

| **Pathway ID** | **Pathway description** | **Count in gene set** | **False discovery rate** |
| --- | --- | --- | --- |
| GO:0044424 | intracellular part | 95 | 1.59e-31 |
| GO:0005622 | intracellular | 95 | 3.54e-31 |
| GO:0005623 | cell | 98 | 3.47e-30 |
| GO:0044464 | cell part | 98 | 3.47e-30 |
| GO:0005737 | cytoplasm | 88 | 5.31e-29 |
| GO:0032991 | macromolecular complex | 50 | 5.43e-25 |
| GO:0044444 | cytoplasmic part | 42 | 1.41e-21 |
| GO:0043232 | intracellular non-membrane-bounded organelle | 39 | 2.18e-20 |
| GO:0005840 | ribosome | 38 | 8.56e-20 |
| GO:0043234 | protein complex | 12 | 5.8e-06 |
| GO:0044391 | ribosomal subunit | 8 | 0.000461 |
| GO:0045259 | proton-transporting ATP synthase complex | 6 | 0.00064 |
| GO:0045261 | proton-transporting ATP synthase complex, catalytic core F(1) | 5 | 0.00064 |
| GO:0015935 | small ribosomal subunit | 4 | 0.0128 |
| GO:0005829 | cytosol | 3 | 0.0155 |
| GO:0005886 | plasma membrane | 9 | 0.0193 |
| GO:0071944 | cell periphery | 9 | 0.0193 |
| GO:0015934 | large ribosomal subunit | 4 | 0.0271 |

**KEGG pathways**

| **Pathway ID** | **Pathway description** | **Count in gene set** | **False discovery rate** |
| --- | --- | --- | --- |
| 03010 | Ribosome | 44 | 8.17e-22 |
| 00970 | Aminoacyl-tRNA biosynthesis | 21 | 4.54e-10 |
| 00230 | Purine metabolism | 31 | 2.08e-08 |
| 01100 | Metabolic pathways | 125 | 1.08e-07 |
| 00240 | Pyrimidine metabolism | 21 | 0.000243 |
| 00550 | Peptidoglycan biosynthesis | 11 | 0.000243 |
| 00190 | Oxidative phosphorylation | 11 | 0.000491 |
| 03018 | RNA degradation | 8 | 0.000522 |
| 01110 | Biosynthesis of secondary metabolites | 55 | 0.000548 |
| 03420 | Nucleotide excision repair | 7 | 0.00181 |
| 00620 | Pyruvate metabolism | 15 | 0.00956 |
| 00480 | Glutathione metabolism | 6 | 0.0323 |
| 01200 | Carbon metabolism | 23 | 0.0323 |
| 00010 | Glycolysis / Gluconeogenesis | 15 | 0.0462 |
| 00061 | Fatty acid biosynthesis | 7 | 0.0462 |
| 00640 | Propanoate metabolism | 7 | 0.0462 |
| 01120 | Microbial metabolism in diverse environments | 35 | 0.0462 |
