# Supplementary material for: A Combined Proteomics, Metabolomics and In Vivo Analysis Approach for the Characterization of Probiotics in Large-Scale Production
Source: Biomolecules. 2020 Jan 18;10(1):157. doi: 10.3390/biom10010157 (PMC7022454; doi:10.3390/biom10010157)
Supplement: Supplementary file 1 [file biomolecules-10-00157-s001.zip › biomolecules-666446--SUPPL/Table S5_ STRING Enrichment analysis B.breve.docx]

**Table S5:** STRING net statistics output and KEGG pathway annotation enrichment analysis of proteins detected more abundant in *Bifidobacterium breve* from US-preparations. PPI and GO/KEGG annotation enrichments were retained significant with a FDR p < 0.001 and p < 0.005 (not shadowed area), respectively.

**Net statistics output**

| number of nodes: | 298 |
| --- | --- |
| number of edges: | 1454 |
| average node degree: | 9.76 |
| avg. local clustering coefficient: | 0.442 |
| expected number of edges: | 1108 |
| PPI enrichment p-value: | < 1.0e-16 |

**KEGG pathways**

| **Pathway ID** | **Pathway description** | **Count in gene set** | **False discovery rate** |
| --- | --- | --- | --- |
| 01100 | Metabolic pathways | 116 | 1.14e-21 |
| 01110 | Biosynthesis of secondary metabolites | 64 | 1.06e-11 |
| 03010 | Ribosome | 28 | 1.1e-08 |
| 00230 | Purine metabolism | 25 | 1.87e-08 |
| 00970 | Aminoacyl-tRNA biosynthesis | 16 | 5.29e-07 |
| 01120 | Microbial metabolism in diverse environments | 32 | 2.6e-06 |
| 01200 | Carbon metabolism | 20 | 2.58e-05 |
| 00520 | Amino sugar and nucleotide sugar metabolism | 14 | 9.87e-05 |
| 00250 | Alanine, aspartate and glutamate metabolism | 11 | 0.000319 |
| 01230 | Biosynthesis of amino acids | 32 | 0.000319 |
| 00240 | Pyrimidine metabolism | 16 | 0.000824 |
| 03018 | RNA degradation | 7 | 0.00149 |
| 00620 | Pyruvate metabolism | 8 | 0.00171 |
| 00010 | Glycolysis / Gluconeogenesis | 11 | 0.00205 |
| 00670 | One carbon pool by folate | 6 | 0.00205 |
| 03020 | RNA polymerase | 4 | 0.00365 |
| 00030 | Pentose phosphate pathway | 8 | 0.00486 |
| 00640 | Propanoate metabolism | 5 | 0.0189 |
| 00350 | Tyrosine metabolism | 3 | 0.0193 |
| 00500 | Starch and sucrose metabolism | 11 | 0.0211 |
| 00190 | Oxidative phosphorylation | 6 | 0.0272 |
| 00521 | Streptomycin biosynthesis | 4 | 0.0304 |
