# Supplementary material for: A Combined Proteomics, Metabolomics and In Vivo Analysis Approach for the Characterization of Probiotics in Large-Scale Production
Source: Biomolecules. 2020 Jan 18;10(1):157. doi: 10.3390/biom10010157 (PMC7022454; doi:10.3390/biom10010157)
Supplement: Supplementary file 1 [file biomolecules-10-00157-s001.zip › biomolecules-666446--SUPPL/Table S6_ STRING Enrichment analysis S.thermophilus.docx]

**Table S6:** STRING net statistics output and GO and KEGG pathway annotation enrichment analysis of proteins detected more abundant in *Streptococcus thermophilus* from US-preparations. PPI and GO/KEGG annotation enrichments were retained significant with a FDR p < 0.001 and p < 0.005 (not shadowed area), respectively.

**Net statistics output**

| number of nodes: | 324 |
| --- | --- |
| number of edges: | 777 |
| average node degree: | 4.8 |
| avg. local clustering coefficient: | 0.371 |
| expected number of edges: | 696 |
| PPI enrichment p-value: | 0.0014 |

**GO BP**

| **Pathway ID** | **Pathway description** | **Count in gene set** | **False discovery rate** |
| --- | --- | --- | --- |
| GO:0008150 | biological_process | 114 | 4.57e-17 |
| GO:0008152 | metabolic process | 111 | 7.26e-17 |
| GO:0009987 | cellular process | 104 | 3.1e-15 |
| GO:0071704 | organic substance metabolic process | 102 | 2.14e-14 |
| GO:0044238 | primary metabolic process | 97 | 1.04e-13 |
| GO:0044237 | cellular metabolic process | 98 | 1.08e-13 |
| GO:0006807 | nitrogen compound metabolic process | 90 | 1.09e-11 |
| GO:0044763 | single-organism cellular process | 66 | 5.59e-11 |
| GO:0044699 | single-organism process | 67 | 3.57e-10 |
| GO:0006725 | cellular aromatic compound metabolic process | 61 | 9.29e-10 |
| GO:0034641 | cellular nitrogen compound metabolic process | 78 | 9.29e-10 |
| GO:1901360 | organic cyclic compound metabolic process | 61 | 1.18e-09 |
| GO:0046483 | heterocycle metabolic process | 59 | 3.69e-09 |
| GO:0006139 | nucleobase-containing compound metabolic process | 55 | 2.05e-08 |
| GO:0044710 | single-organism metabolic process | 57 | 4.8e-08 |
| GO:1901576 | organic substance biosynthetic process | 68 | 7.87e-08 |
| GO:0044281 | small molecule metabolic process | 46 | 1.42e-07 |
| GO:1901564 | organonitrogen compound metabolic process | 63 | 1.57e-07 |
| GO:0044249 | cellular biosynthetic process | 66 | 2.54e-07 |
| GO:0044711 | single-organism biosynthetic process | 39 | 2.56e-07 |
| GO:0043170 | macromolecule metabolic process | 63 | 2.83e-07 |
| GO:0044260 | cellular macromolecule metabolic process | 60 | 8.53e-07 |
| GO:1901566 | organonitrogen compound biosynthetic process | 55 | 3.63e-06 |
| GO:0090304 | nucleic acid metabolic process | 34 | 8.03e-05 |
| GO:0071840 | cellular component organization or biogenesis | 18 | 0.000151 |
| GO:1901135 | carbohydrate derivative metabolic process | 25 | 0.000151 |
| GO:0006082 | organic acid metabolic process | 27 | 0.000176 |
| GO:0044271 | cellular nitrogen compound biosynthetic process | 48 | 0.000204 |
| GO:0046128 | purine ribonucleoside metabolic process | 14 | 0.000288 |
| GO:0009119 | ribonucleoside metabolic process | 18 | 0.000336 |
| GO:0019752 | carboxylic acid metabolic process | 26 | 0.000356 |
| GO:0006396 | RNA processing | 15 | 0.000422 |
| GO:0006796 | phosphate-containing compound metabolic process | 25 | 0.000422 |
| GO:0016053 | organic acid biosynthetic process | 18 | 0.000422 |
| GO:0019438 | aromatic compound biosynthetic process | 27 | 0.000422 |
| GO:0019637 | organophosphate metabolic process | 23 | 0.000437 |
| GO:0055086 | nucleobase-containing small molecule metabolic process | 23 | 0.000581 |
| GO:0072521 | purine-containing compound metabolic process | 15 | 0.000581 |
| GO:1901362 | organic cyclic compound biosynthetic process | 27 | 0.00067 |
| GO:0034660 | ncRNA metabolic process | 19 | 0.000989 |
| GO:0034470 | ncRNA processing | 14 | 0.000993 |
| GO:0046394 | carboxylic acid biosynthetic process | 17 | 0.000993 |
| GO:0016070 | RNA metabolic process | 24 | 0.00102 |
| GO:0009167 | purine ribonucleoside monophosphate metabolic process | 12 | 0.00108 |
| GO:0065007 | biological regulation | 18 | 0.00109 |
| GO:0010467 | gene expression | 39 | 0.00132 |
| GO:0006163 | purine nucleotide metabolic process | 14 | 0.00145 |
| GO:0009150 | purine ribonucleotide metabolic process | 13 | 0.0016 |
| GO:0018130 | heterocycle biosynthetic process | 25 | 0.0016 |
| GO:1901137 | carbohydrate derivative biosynthetic process | 20 | 0.00179 |
| GO:0019693 | ribose phosphate metabolic process | 16 | 0.00221 |
| GO:0009117 | nucleotide metabolic process | 20 | 0.00234 |
| GO:0050794 | regulation of cellular process | 17 | 0.00241 |
| GO:0016043 | cellular component organization | 12 | 0.00255 |
| GO:0019538 | protein metabolic process | 33 | 0.00255 |
| GO:0043412 | macromolecule modification | 12 | 0.00255 |
| GO:0006399 | tRNA metabolic process | 16 | 0.00267 |
| GO:0008033 | tRNA processing | 11 | 0.00267 |
| GO:0042455 | ribonucleoside biosynthetic process | 14 | 0.00267 |
| GO:0046129 | purine ribonucleoside biosynthetic process | 10 | 0.00267 |
| GO:0009259 | ribonucleotide metabolic process | 15 | 0.00321 |
| GO:0008652 | cellular amino acid biosynthetic process | 14 | 0.00392 |
| GO:1901575 | organic substance catabolic process | 11 | 0.00408 |
| GO:0046034 | ATP metabolic process | 8 | 0.00429 |
| GO:0009161 | ribonucleoside monophosphate metabolic process | 13 | 0.00449 |
| GO:0044085 | cellular component biogenesis | 13 | 0.00449 |
| GO:0043648 | dicarboxylic acid metabolic process | 6 | 0.00517 |
| GO:0009141 | nucleoside triphosphate metabolic process | 10 | 0.00691 |
| GO:0034654 | nucleobase-containing compound biosynthetic process | 21 | 0.00691 |
| GO:0090305 | nucleic acid phosphodiester bond hydrolysis | 10 | 0.00691 |
| GO:0090407 | organophosphate biosynthetic process | 17 | 0.00691 |
| GO:0009199 | ribonucleoside triphosphate metabolic process | 9 | 0.00752 |
| GO:0044267 | cellular protein metabolic process | 29 | 0.00958 |
| GO:1901607 | alpha-amino acid biosynthetic process | 12 | 0.0107 |
| GO:0006810 | transport | 9 | 0.0111 |
| GO:0034655 | nucleobase-containing compound catabolic process | 6 | 0.0111 |
| GO:0006400 | tRNA modification | 8 | 0.0119 |
| GO:0009168 | purine ribonucleoside monophosphate biosynthetic process | 8 | 0.0119 |
| GO:0072524 | pyridine-containing compound metabolic process | 7 | 0.012 |
| GO:0006520 | cellular amino acid metabolic process | 18 | 0.0161 |
| GO:0005975 | carbohydrate metabolic process | 9 | 0.0162 |
| GO:0006457 | protein folding | 5 | 0.0162 |
| GO:0006464 | cellular protein modification process | 5 | 0.0162 |
| GO:0009152 | purine ribonucleotide biosynthetic process | 9 | 0.0162 |
| GO:0009451 | RNA modification | 9 | 0.0162 |
| GO:0034645 | cellular macromolecule biosynthetic process | 34 | 0.0162 |
| GO:0043650 | dicarboxylic acid biosynthetic process | 5 | 0.0162 |
| GO:0044265 | cellular macromolecule catabolic process | 5 | 0.0162 |
| GO:0009165 | nucleotide biosynthetic process | 14 | 0.019 |
| GO:0032787 | monocarboxylic acid metabolic process | 7 | 0.019 |
| GO:0044255 | cellular lipid metabolic process | 6 | 0.019 |
| GO:0055085 | transmembrane transport | 7 | 0.019 |
| GO:0098660 | inorganic ion transmembrane transport | 6 | 0.019 |
| GO:0006732 | coenzyme metabolic process | 9 | 0.0219 |
| GO:0010468 | regulation of gene expression | 9 | 0.0219 |
| GO:0065008 | regulation of biological quality | 9 | 0.0219 |
| GO:0080090 | regulation of primary metabolic process | 10 | 0.0251 |
| GO:0044765 | single-organism transport | 8 | 0.0253 |
| GO:0009260 | ribonucleotide biosynthetic process | 11 | 0.027 |
| GO:0006091 | generation of precursor metabolites and energy | 5 | 0.0285 |
| GO:0006167 | AMP biosynthetic process | 3 | 0.0285 |
| GO:0009089 | lysine biosynthetic process via diaminopimelate | 3 | 0.0285 |
| GO:0016071 | mRNA metabolic process | 3 | 0.0285 |
| GO:0018193 | peptidyl-amino acid modification | 3 | 0.0285 |
| GO:0019877 | diaminopimelate biosynthetic process | 3 | 0.0285 |
| GO:0046496 | nicotinamide nucleotide metabolic process | 6 | 0.0285 |
| GO:0090501 | RNA phosphodiester bond hydrolysis | 5 | 0.0285 |
| GO:2000112 | regulation of cellular macromolecule biosynthetic process | 9 | 0.0285 |
| GO:0009156 | ribonucleoside monophosphate biosynthetic process | 9 | 0.0385 |
| GO:0006364 | rRNA processing | 5 | 0.0485 |
| GO:0008610 | lipid biosynthetic process | 5 | 0.0485 |
| GO:0042777 | plasma membrane ATP synthesis coupled proton transport | 4 | 0.0485 |
| GO:0043173 | nucleotide salvage | 4 | 0.0485 |
| GO:0043174 | nucleoside salvage | 4 | 0.0485 |
| GO:0043603 | cellular amide metabolic process | 26 | 0.0485 |
| GO:0051171 | regulation of nitrogen compound metabolic process | 9 | 0.0485 |

**GO MF**

| **Pathway ID** | **Pathway description** | **Count in gene set** | **False discovery rate** |
| --- | --- | --- | --- |
| GO:0003674 | molecular_function | 110 | 3.04e-16 |
| GO:0003824 | catalytic activity | 89 | 3.04e-16 |
| GO:0043167 | ion binding | 66 | 3.57e-13 |
| GO:0005488 | binding | 84 | 8.66e-13 |
| GO:0046872 | metal ion binding | 38 | 4.44e-10 |
| GO:0097159 | organic cyclic compound binding | 70 | 4.44e-10 |
| GO:1901363 | heterocyclic compound binding | 70 | 4.44e-10 |
| GO:0000166 | nucleotide binding | 49 | 1.4e-09 |
| GO:0043168 | anion binding | 47 | 4.11e-09 |
| GO:0032550 | purine ribonucleoside binding | 42 | 8.84e-08 |
| GO:0032555 | purine ribonucleotide binding | 42 | 8.84e-08 |
| GO:0035639 | purine ribonucleoside triphosphate binding | 42 | 8.84e-08 |
| GO:0016787 | hydrolase activity | 33 | 1.65e-07 |
| GO:0005524 | ATP binding | 34 | 1.47e-05 |
| GO:0000287 | magnesium ion binding | 15 | 0.000115 |
| GO:0016740 | transferase activity | 28 | 0.000121 |
| GO:0016462 | pyrophosphatase activity | 16 | 0.000177 |
| GO:0017111 | nucleoside-triphosphatase activity | 15 | 0.000182 |
| GO:0046914 | transition metal ion binding | 16 | 0.000712 |
| GO:0016874 | ligase activity | 16 | 0.00722 |
| GO:0016788 | hydrolase activity, acting on ester bonds | 12 | 0.00786 |
| GO:0004518 | nuclease activity | 10 | 0.0104 |
| GO:0004527 | exonuclease activity | 5 | 0.0107 |
| GO:0042625 | ATPase activity, coupled to transmembrane movement of ions | 6 | 0.0179 |
| GO:0005525 | GTP binding | 8 | 0.0182 |
| GO:0016887 | ATPase activity | 8 | 0.0182 |
| GO:0042623 | ATPase activity, coupled | 7 | 0.0182 |
| GO:0016879 | ligase activity, forming carbon-nitrogen bonds | 9 | 0.0256 |
| GO:0008270 | zinc ion binding | 9 | 0.0347 |
| GO:0004540 | ribonuclease activity | 5 | 0.0432 |
| GO:0008080 | N-acetyltransferase activity | 3 | 0.0432 |
| GO:0008408 | 3 -5 exonuclease activity | 3 | 0.0432 |
| GO:0016747 | transferase activity, transferring acyl groups other than amino-acyl groups | 5 | 0.0432 |
| GO:0016796 | exonuclease activity, active with either ribo- or deoxyribonucleic acids and producing 5 -phosphomonoesters | 3 | 0.0432 |
| GO:0046961 | proton-transporting ATPase activity, rotational mechanism |  |  |

**GO CC**

| **Pathway ID** | **Pathway description** | **Count in gene set** | **False discovery rate** |
| --- | --- | --- | --- |
| GO:0005623 | cell | 81 | 8.47e-11 |
| GO:0044464 | cell part | 81 | 8.47e-11 |
| GO:0005622 | intracellular | 75 | 1.54e-10 |
| GO:0044424 | intracellular part | 74 | 2.91e-10 |
| GO:0005737 | cytoplasm | 69 | 7.2e-09 |
| GO:0043234 | protein complex | 12 | 0.00103 |
| GO:0098796 | membrane protein complex | 7 | 0.0112 |
| GO:0016020 | membrane | 12 | 0.0114 |
| GO:1902494 | catalytic complex | 8 | 0.0149 |
| GO:0032991 | macromolecular complex | 23 | 0.0158 |
| GO:0045261 | proton-transporting ATP synthase complex, catalytic core F(1) | 4 | 0.017 |
| GO:0005886 | plasma membrane | 11 | 0.0173 |
| GO:0071944 | cell periphery | 11 | 0.0173 |
| GO:0045259 | proton-transporting ATP synthase complex | 4 | 0.0338 |
| GO:0044425 | membrane part | 9 | 0.0454 |

**KEGG pathways**

| **Pathway ID** | **Pathway description** | **Count in gene set** | **False discovery rate** |
| --- | --- | --- | --- |
| 01100 | Metabolic pathways | 101 | 3.35e-11 |
| 01230 | Biosynthesis of amino acids | 35 | 4.29e-05 |
| 01110 | Biosynthesis of secondary metabolites | 46 | 0.000518 |
| 01120 | Microbial metabolism in diverse environments | 27 | 0.00233 |
| 01200 | Carbon metabolism | 19 | 0.00233 |
| 00620 | Pyruvate metabolism | 11 | 0.00312 |
| 00061 | Fatty acid biosynthesis | 7 | 0.00762 |
| 01212 | Fatty acid metabolism | 7 | 0.00762 |
| 00473 | D-Alanine metabolism | 4 | 0.00797 |
| 00020 | Citrate cycle (TCA cycle) | 5 | 0.0189 |
