# Supplementary material for: A Combined Proteomics, Metabolomics and In Vivo Analysis Approach for the Characterization of Probiotics in Large-Scale Production
Source: Biomolecules. 2020 Jan 18;10(1):157. doi: 10.3390/biom10010157 (PMC7022454; doi:10.3390/biom10010157)
Supplement: Supplementary file 1 [file biomolecules-10-00157-s001.zip › biomolecules-666446--SUPPL/Table S7_STRING Enrichment analysis L.plantarum.docx]

**Table S7:** STRING net statistics output and GO and KEGG pathway annotation enrichment analysis of proteins detected more abundant in *Lactobacillus plantarum* from US-preparations. PPI and GO/KEGG annotation enrichments were retained significant with a FDR p < 0.001 and p < 0.005 (not shadowed area), respectively.

**Net statistics output**

| number of nodes: | 54 |
| --- | --- |
| number of edges: | 226 |
| average node degree: | 8.37 |
| avg. local clustering coefficient: | 0.582 |
| expected number of edges: | 118 |
| PPI enrichment p-value: | < 1.0e-16 |

**GO BP**

| **Pathway ID** | **Pathway description** | **Count in gene set** | **False discovery rate** |
| --- | --- | --- | --- |
| GO:0044267 | cellular protein metabolic process | 21 | 3.82e-14 |
| GO:0006412 | translation | 18 | 1.14e-12 |
| GO:1901564 | organonitrogen compound metabolic process | 25 | 1.14e-12 |
| GO:0044238 | primary metabolic process | 28 | 4.47e-11 |
| GO:0034641 | cellular nitrogen compound metabolic process | 25 | 4.8e-11 |
| GO:0044237 | cellular metabolic process | 28 | 5.62e-11 |
| GO:0008152 | metabolic process | 29 | 1.25e-10 |
| GO:0071704 | organic substance metabolic process | 28 | 1.25e-10 |
| GO:0010467 | gene expression | 18 | 1.06e-09 |
| GO:0044271 | cellular nitrogen compound biosynthetic process | 19 | 5.59e-09 |
| GO:1901576 | organic substance biosynthetic process | 22 | 5.94e-09 |
| GO:1901566 | organonitrogen compound biosynthetic process | 19 | 1.61e-08 |
| GO:0006096 | glycolytic process | 6 | 1.46e-07 |
| GO:0009161 | ribonucleoside monophosphate metabolic process | 7 | 6.18e-05 |
| GO:0009119 | ribonucleoside metabolic process | 7 | 9.79e-05 |
| GO:0009259 | ribonucleotide metabolic process | 7 | 0.000125 |
| GO:0006094 | gluconeogenesis | 3 | 0.0003 |
| GO:0006414 | translational elongation | 3 | 0.00129 |
| GO:0006457 | protein folding | 3 | 0.00666 |
| GO:0019752 | carboxylic acid metabolic process | 7 | 0.0224 |

**GO MF**

| **Pathway ID** | **Pathway description** | **Count in gene set** | **False discovery rate** |
| --- | --- | --- | --- |
| GO:0003735 | structural constituent of ribosome | 14 | 1.88e-11 |
| GO:0003674 | molecular_function | 29 | 4.83e-10 |
| GO:0003723 | RNA binding | 14 | 1.23e-09 |
| GO:0019843 | rRNA binding | 11 | 2.1e-09 |
| GO:0097159 | organic cyclic compound binding | 20 | 4.87e-07 |
| GO:1901363 | heterocyclic compound binding | 20 | 4.87e-07 |
| GO:0005488 | binding | 21 | 8.26e-07 |
| GO:0003746 | translation elongation factor activity | 3 | 0.00614 |
| GO:0004457 | lactate dehydrogenase activity | 2 | 0.0481 |
| GO:0016853 | isomerase activity | 4 | 0.0481 |

**GO CC**

| **Pathway ID** | **Pathway description** | **Count in gene set** | **False discovery rate** |
| --- | --- | --- | --- |
| GO:0005622 | intracellular | 25 | 1.37e-12 |
| GO:0005737 | cytoplasm | 25 | 1.37e-12 |
| GO:0005840 | ribosome | 14 | 1.37e-12 |
| GO:0044444 | cytoplasmic part | 15 | 1.37e-12 |
| GO:0005623 | cell | 25 | 3.75e-11 |
| GO:0032991 | macromolecular complex | 15 | 4.02e-11 |
| GO:0044391 | ribosomal subunit | 3 | 0.00913 |
| GO:0015935 | small ribosomal subunit | 2 | 0.0275 |

**KEGG pathways**

| **Pathway ID** | **Pathway description** | **Count in gene set** | **False discovery rate** |
| --- | --- | --- | --- |
| 03010 | Ribosome | 15 | 2.9e-13 |
| 00010 | Glycolysis / Gluconeogenesis | 9 | 6.54e-06 |
| 01200 | Carbon metabolism | 9 | 9.72e-05 |
| 01120 | Microbial metabolism in diverse environments | 11 | 0.000458 |
| 00480 | Glutathione metabolism | 3 | 0.0153 |
| 01110 | Biosynthesis of secondary metabolites | 11 | 0.0166 |
| 03018 | RNA degradation | 3 | 0.0166 |
| 01230 | Biosynthesis of amino acids | 7 | 0.0383 |
