# Supplementary material for: A Combined Proteomics, Metabolomics and In Vivo Analysis Approach for the Characterization of Probiotics in Large-Scale Production
Source: Biomolecules. 2020 Jan 18;10(1):157. doi: 10.3390/biom10010157 (PMC7022454; doi:10.3390/biom10010157)
Supplement: Supplementary file 1 [file biomolecules-10-00157-s001.zip › biomolecules-666446--SUPPL/Table S8_STRING Enrichment analysis L.acidophilus.docx]

**Table S8:** STRING net statistics output and GO and KEGG pathway annotation enrichment analysis of proteins detected more abundant in *Lactobacillus acidophilus* from IT-preparations. PPI and GO/KEGG annotation enrichments were retained significant with a FDR p < 0.001 and p < 0.005 (not shadowed area), respectively.

**Net statistics output**

| number of nodes: | 73 |
| --- | --- |
| number of edges: | 77 |
| average node degree: | 2.11 |
| avg. local clustering coefficient: | 0.354 |
| expected number of edges: | 56 |
| PPI enrichment p-value: | 0.00435 |

**GO BP**

| **Pathway ID** | **Pathway description** | **Count in gene set** | **False discovery rate** |
| --- | --- | --- | --- |
| GO:0006412 | translation | 11 | 0.018 |
| GO:0019538 | protein metabolic process | 13 | 0.018 |
| GO:0034645 | cellular macromolecule biosynthetic process | 14 | 0.018 |
| GO:0044267 | cellular protein metabolic process | 12 | 0.018 |
| GO:1901566 | organonitrogen compound biosynthetic process | 15 | 0.018 |
| GO:0044249 | cellular biosynthetic process | 16 | 0.0189 |
| GO:1901576 | organic substance biosynthetic process | 16 | 0.0198 |
| GO:0043170 | macromolecule metabolic process | 16 | 0.0263 |
| GO:0071704 | organic substance metabolic process | 20 | 0.0263 |
| GO:0010467 | gene expression | 12 | 0.0457 |
| GO:0044260 | cellular macromolecule metabolic process | 15 | 0.0468 |
| GO:0044271 | cellular nitrogen compound biosynthetic process | 13 | 0.0468 |

**GO MF**

| **Pathway ID** | **Pathway description** | **Count in gene set** | **False discovery rate** |
| --- | --- | --- | --- |
| GO:0003735 | structural constituent of ribosome | 9 | 0.0171 |

**GO CC**

| **Pathway ID** | **Pathway description** | **Count in gene set** | **False discovery rate** |
| --- | --- | --- | --- |
| GO:0043232 | intracellular non-membrane-bounded organelle | 10 | 0.000226 |
| GO:0005840 | ribosome | 9 | 0.000869 |
| GO:0005737 | cytoplasm | 16 | 0.00325 |
| GO:0005622 | intracellular | 16 | 0.00481 |
| GO:0005623 | cell | 16 | 0.0169 |

**KEGG pathways**

| **Pathway ID** | **Pathway description** | **Count in gene set** | **False discovery rate** |
| --- | --- | --- | --- |
| 03010 | Ribosome | 10 | 0.00188 |
| 00473 | D-Alanine metabolism | 3 | 0.0092 |
| 00620 | Pyruvate metabolism | 5 | 0.0215 |
