# Supplementary material for: A Combined Proteomics, Metabolomics and In Vivo Analysis Approach for the Characterization of Probiotics in Large-Scale Production
Source: Biomolecules. 2020 Jan 18;10(1):157. doi: 10.3390/biom10010157 (PMC7022454; doi:10.3390/biom10010157)
Supplement: Supplementary file 1 [file biomolecules-10-00157-s001.zip › biomolecules-666446--SUPPL/Table S9_STRING Enrichment analysis B.longum & B.infantis.docx]

**Table S9:** STRING net statistics output and GO and KEGG pathway annotation enrichment analysis of proteins detected more abundant in *Bifidobacterium longum* and *Bifidobacterium infantis* from US-preparations. *B. longum* was set as reference organism. PPI and GO/KEGG annotation enrichments were retained significant with a FDR p < 0.001 and p < 0.005 (not shadowed area), respectively.

**Net statistics output**

| number of nodes: | 83 |
| --- | --- |
| number of edges: | 292 |
| average node degree: | 7.04 |
| avg. local clustering coefficient: | 0.475 |
| expected number of edges: | 196 |
| PPI enrichment p-value: | 1.09e-10 |

**GO BP terms**

| **Pathway ID** | **Pathway description** | **Count in gene set** | **False discovery rate** |
| --- | --- | --- | --- |
| GO:0043604 | amide biosynthetic process | 28 | 1.82e-15 |
| GO:0006412 | translation | 27 | 2.47e-15 |
| GO:0044267 | cellular protein metabolic process | 29 | 2.47e-15 |
| GO:1901564 | organonitrogen compound metabolic process | 38 | 2.47e-15 |
| GO:0044271 | cellular nitrogen compound biosynthetic process | 34 | 2.81e-15 |
| GO:1901566 | organonitrogen compound biosynthetic process | 36 | 4.18e-15 |
| GO:0034641 | cellular nitrogen compound metabolic process | 39 | 1.17e-14 |
| GO:0044237 | cellular metabolic process | 43 | 3.16e-14 |
| GO:0006807 | nitrogen compound metabolic process | 40 | 1.9e-13 |
| GO:1901576 | organic substance biosynthetic process | 37 | 1.9e-13 |
| GO:0071704 | organic substance metabolic process | 43 | 1.93e-13 |
| GO:0044249 | cellular biosynthetic process | 36 | 5.79e-13 |
| GO:0044238 | primary metabolic process | 40 | 6.38e-12 |
| GO:0010467 | gene expression | 27 | 1.39e-11 |
| GO:0044260 | cellular macromolecule metabolic process | 31 | 1.84e-11 |
| GO:0044281 | small molecule metabolic process | 15 | 0.00625 |
| GO:0006732 | coenzyme metabolic process | 6 | 0.0114 |
| GO:0042364 | water-soluble vitamin biosynthetic process | 3 | 0.0234 |
| GO:0044710 | single-organism metabolic process | 16 | 0.0234 |
| GO:0006725 | cellular aromatic compound metabolic process | 15 | 0.0421 |
| GO:0019637 | organophosphate metabolic process | 9 | 0.0429 |
| GO:0046483 | heterocycle metabolic process | 15 | 0.0429 |
| GO:0009108 | coenzyme biosynthetic process | 4 | 0.0472 |
| GO:0019752 | carboxylic acid metabolic process | 10 | 0.0472 |
| GO:1901360 | organic cyclic compound metabolic process | 15 | 0.0472 |
| GO:0044763 | single-organism cellular process | 16 | 0.049 |

**GO MF terms**

| **Pathway ID** | **Pathway description** | **Count in gene set** | **False discovery rate** |
| --- | --- | --- | --- |
| GO:0003735 | structural constituent of ribosome | 20 | 1.45e-12 |
| GO:0003674 | molecular_function | 43 | 8.17e-12 |
| GO:0003723 | RNA binding | 20 | 4.5e-11 |
| GO:0019843 | rRNA binding | 16 | 9.85e-11 |
| GO:0005488 | binding | 35 | 1.1e-10 |
| GO:0097159 | organic cyclic compound binding | 32 | 1.1e-10 |
| GO:1901363 | heterocyclic compound binding | 32 | 1.1e-10 |
| GO:0003676 | nucleic acid binding | 22 | 2.65e-09 |
| GO:0000049 | tRNA binding | 6 | 0.00104 |
| GO:0017076 | purine nucleotide binding | 13 | 0.0119 |
| GO:0016829 | lyase activity | 5 | 0.0211 |
| GO:0032550 | purine ribonucleoside binding | 12 | 0.0211 |
| GO:0032555 | purine ribonucleotide binding | 12 | 0.0211 |
| GO:0035639 | purine ribonucleoside triphosphate binding | 12 | 0.0211 |
| GO:0043167 | ion binding | 16 | 0.0211 |
| GO:0016874 | ligase activity | 7 | 0.0267 |
| GO:0003743 | translation initiation factor activity | 2 | 0.0386 |
| GO:0003824 | catalytic activity | 19 | 0.0398 |

**GO CC terms**

| **Pathway ID** | **Pathway description** | **Count in gene set** | **False discovery rate** |
| --- | --- | --- | --- |
| GO:0005840 | ribosome | 20 | 9.95e-14 |
| GO:0005737 | cytoplasm | 33 | 1.5e-12 |
| GO:0005622 | intracellular | 33 | 4.69e-12 |
| GO:0005623 | cell | 33 | 1.29e-10 |
| GO:0044391 | ribosomal subunit | 7 | 4.18e-06 |
| GO:0015935 | small ribosomal subunit | 5 | 1.45e-05 |

**KEGG pathways**

| **Pathway ID** | **Pathway description** | **Count in gene set** | **False discovery rate** |
| --- | --- | --- | --- |
| 03010 | Ribosome | 21 | 8.35e-14 |
| 01100 | Metabolic pathways | 29 | 0.00263 |
| 00970 | Aminoacyl-tRNA biosynthesis | 6 | 0.0159 |
